# Simulating Visibility and Reading Performance in Low Vision

**DOI:** 10.3389/fnins.2021.671121

**Published:** 2021-07-05

**Authors:** Ying-Zi Xiong, Quan Lei, Aurélie Calabrèse, Gordon E. Legge

**Affiliations:** ^1^Department of Psychology, University of Minnesota, Minneapolis, MN, United States; ^2^Department of Psychology, Wichita State University, Wichita, KS, United States; ^3^Inria, Université Côte d’Azur, Sophia Antipolis, France

**Keywords:** reading, low vision, text visibility, visual acuity, contrast sensitivity

## Abstract

**Purpose:**

Low vision reduces text visibility and causes difficulties in reading. A valid low-vision simulation could be used to evaluate the accessibility of digital text for readers with low vision. We examined the validity of a digital simulation for replicating the text visibility and reading performance of low-vision individuals.

**Methods:**

Low-vision visibility was modeled with contrast sensitivity functions (CSFs) with parameters to represent reduced acuity and contrast sensitivity. Digital filtering incorporating these CSFs were applied to digital versions of the Lighthouse Letter Acuity Chart and the Pelli-Robson Contrast Sensitivity Chart. Reading performance (reading acuity, critical print size, and maximum reading speed) was assessed with filtered versions of the MNREAD reading acuity Chart. Thirty-six normally sighted young adults completed chart testing under normal and simulated low-vision conditions. Fifty-eight low-vision subjects (thirty with macular pathology and twenty-eight with non-macular pathology) and fifteen normally sighted older subjects completed chart testing with their habitual viewing. We hypothesized that the performance of the normally sighted young adults under simulated low-vision conditions would match the corresponding performance of actual low-vision subjects.

**Results:**

When simulating low-vision conditions with visual acuity better than 1.50 logMAR (Snellen 20/630) and contrast sensitivity better than 0.15 log unit, the simulation adequately reduced the acuity and contrast sensitivity in normally sighted young subjects to the desired low-vision levels. When performing the MNREAD test with simulated low vision, the normally sighted young adults had faster maximum reading speed than both the Non-macular and Macular groups, by an average of 0.07 and 0.12 log word per minute, respectively. However, they adequately replicated the reading acuity as well as the critical print size, up to 2.00 logMAR of both low-vision groups.

**Conclusion:**

A low-vision simulation based on clinical measures of visual acuity and contrast sensitivity can provide good estimates of reading performance and the accessibility of digital text for a broad range of low-vision conditions.

## Introduction

Low vision refers to any vision impairment that cannot be corrected by glasses or contact lenses. For readers with low vision, text legibility is limited by acuity and contrast sensitivity. In practical terms, reduced acuity and contrast sensitivity limit the ability to see graphics and text on web pages and in other digital formats. Other factors affecting vision, such as field loss, light level and glare, often add to the difficulties in low-vision function (Fletcher et al., 1999; Turano et al., 2004; Kiser et al., 2005). While it is not always sufficient for successful low-vision functioning, the visibility of key features is usually a necessary condition for low-vision functioning. The goal of our project is to validate a simulation of the loss of visibility due to reduced acuity and reduced contrast sensitivity. The simulation is based on image filtering that uses transformations of the normal contrast sensitivity function (CSF) to represent reduced visibility associated with low vision. We evaluated the validity of the simulation by testing normally sighted subjects on filtered images of text to determine if measures of acuity, contrast sensitivity and reading performance match the performance of people with actual low vision. A valid simulation of low-vision visibility could be useful to eye-care clinicians, display designers, website creators, and family members in evaluating the accessibility of digital rendering of text or graphics for people with low vision.

Low-vision simulations, such as diffusive filters, optical defocus and digital blur, have been utilized for research or education purposes (Peli, 1990; Dickinson and Rabbitt, 1991; Bowers and Reid, 1997; Thompson et al., 2017; Jones and Ometto, 2018; Jones et al., 2020). A desirable property of an digital simulation is that it can be parameterized by measurable properties of vision status such as acuity and contrast sensitivity (Peli, 1990; Thompson et al., 2017).

The CSF is a detailed measurement of an individual’s acuity limit and contrast sensitivity across a range of spatial frequencies (Campbell and Robson, 1968), which determines the visibility of any pattern. Compared to people with normal vision, people with low vision often have reduced contrast sensitivity and a decreased range of visible spatial frequencies (Ross et al., 1984; Sokol et al., 1985; Chylack et al., 1993). Peli described a methodology using low-vision CSF filters to process images to represent the reduction in sensitivity of low-vision eyes (Peli, 1990). A key assumption of the method is that target features in the original image that are not visible or recognizable with specific levels of low vision are not visible or recognizable to normally sighted subjects viewing the filtered image.

It is difficult in practice to directly measure CSFs for people with low vision, although recent development of a quick CSF measurement facilitates such measurement (Lesmes et al., 2010; Elfadaly et al., 2020). Another approach is to derive low-vision CSFs from a typical CSF for normal vision. Chung and Legge (2016) proposed that low vision CSFs can be approximated by horizontal and/or vertical scaling of a normal vision CSF template, with the horizontal scaling representing the loss in high spatial frequency resolution, and the vertical scaling representing the loss in peak contrast sensitivity (Chung and Legge, 2016).

Recent studies have further shown that the horizontal and vertical scaling factors for deriving the low-vision CSF can be estimated by clinical measures of visual acuity and contrast sensitivity (Thurman et al., 2016; Thompson et al., 2017). Specifically, clinical testing tools such as letter acuity charts [e.g., the Early Treatment of Diabetic Retinopathy (ETDRS) chart] and letter contrast sensitivity charts (e.g., the Pelli-Robson Chart), were designed to provide convenient measures of individual visual acuity and contrast sensitivity. These measures provide reasonable estimations of the high spatial frequency resolution and the peak contrast sensitivity of the individual’s CSF curve (Thurman et al., 2016; Thompson et al., 2017). Using the filtering method proposed by Peli (1990), Thompson and colleagues (Thompson et al., 2017) parameterized their low-vision filters using these clinical measures in an attempt to simulate visibility experienced by individuals with reduced acuity and contrast sensitivity. Their simulation was validated by a letter recognition task, showing that the measured acuity for filtered letters closely matched their intended visibility as specified by the filter parameters. Despite the potential usefulness of the method, it is unknown whether the method can also be used to simulate the impact of reduced visibility on more complex tasks such as reading.

A primary goal of the current study was to examine the validity of the CSF filtering method for predicting visual performance in a task beyond simple visibility. We simulated the reading performance of people with low vision. Following Peli (1990) and Thompson et al. (2017), we embedded an estimate of the reader’s CSF in the simulation filter. The implementation included two key steps: (1) clinical acuity and contrast sensitivity measured by letter charts were used to estimate the scaling factors used to derive the low-vision CSF; and (2) the low-vision CSF thus derived was used to filter the input image to generate the simulation.

To summarize, the current study was aimed to extend previous work by using clinical measures of acuity and contrast sensitivity to parameterize the simulation method and to systematically validate the method by examining the impact of simulated low vision on both simple tasks such as letter recognition and complex tasks such as reading. Specifically, we asked two main questions: (1) Do normally sighted subjects tested with filtered images of the letter charts show reduced acuity and contrast sensitivity close to the simulated low-vision levels? And (2) Do the reduced acuity and contrast sensitivity have the same impact on reading as real low vision? To this end, we compared the reading performance of normally sighted subjects, tested with simulated reduction of acuity and contrast sensitivity, with the performance of low-vision subjects with the equivalent acuity and contrast sensitivity. We also examined whether two other factors beyond acuity and contrast sensitivity, namely age and central vision status, need to be considered in the simulation. It has been well studied that people with central field loss due to macular diseases have greater difficulty in reading (Legge et al., 1992), therefore we included low-vision groups with non-macular and macular diseases, to compare the validity of our simulation for low vision with or without central vision disturbance. We included a group of normally sighted older subjects, to examine the need for age adjustment when simulating older low-vision individuals.

## Materials and Methods

### Subjects

One hundred and nine subjects participated in this study. All subjects were native English speakers with no known visual reading disabilities. Normal cognitive status was verified by the Mini-Mental State Examination (score > 24). All subjects were tested with their most up-to-date reading glasses, if any.

Thirty-six of the subjects were normally sighted young adults (YN, 20.5 ± 3.6 years) recruited from the University of Minnesota. Fifteen of the subjects were normally sighted older adults (ON, 68.0 ± 5.0 years) recruited from the Retiree Volunteer Center at the University of Minnesota. Fifty-eight of the subjects (64.8 ± 18.0 years) were adults with low vision whose data were included from two published studies (Cheong et al., 2008; Calabrèse et al., 2018). The low-vision data were separated into macular disease (Mac, *n* = 30) and non-macular disease (Non-Mac, *n* = 28) groups based on whether the diagnoses primarily affected the macular area (see Supplementary Appendix 2 for individual diagnoses). This study was approved by the University of Minnesota Institutional Review Board and followed the Declaration of Helsinki. Consent forms were acquired from all subjects prior to their participation.

### Apparatus and Stimuli

Digital versions of the Lighthouse Letter Acuity Chart, Pelli-Robson Contrast Sensitivity Chart and MNREAD Chart were adapted from the original printed charts (Ferris et al., 1982; Pelli et al., 1988; Mansfield and Legge, 2007), using Psychtoolbox 3.0 software (Pelli, 1997) with Matlab R2016a. In the digital acuity test, a group of five letters was presented on the screen each time, equivalent to a single line on the printed chart. In the digital contrast sensitivity test, a group of three letters was presented on the screen each time, equivalent to a single contrast level on the printed chart. The MNREAD sentences were created by a MNREAD sentence generator (Mansfield et al., 2019). Each MNREAD chart had 21 sentences with decreasing sizes in 0.1 log unit steps from 1.7 logMAR to −0.3 logMAR (equivalent to a range of x-heights from 4.18 to 0.04 degree). Each sentence was formatted on three equal-length lines like the printed MNREAD chart. Only one sentence was presented on the screen at one time.

A large LCD monitor was used (dimensions = 59.6 × 33.4 cm) to ensure the presentation of large size letters (Cinema Display, Apple, Inc.). The refresh rate was 60 Hz and the resolution was 2,560 × 1,440. Stimuli were displayed with 14-bit grayscale resolution using Bits++ (Cambridge Research Systems Ltd., United Kingdom). The output luminance of the monitor at each gray level was measured using a photometer (PR655 Spectroradiometer, Photo Research Inc.), and a look-up table was created to present letters at each contrast level. The white background had a fixed luminance of 298.5 cd/m^2^. For the Lighthouse Letter Acuity Chart and MNREAD Chart, the high-contrast black letters had a fixed luminance of 1.5 cd/m^2^. For the Pelli-Robson Contrast Sensitivity Chart, the luminance of the sixteen three-letter groups ranged from 1.5 to 296.8 cd/m^2^.

The viewing distance was 100 cm, with the exception that the small print sizes (<0 logMAR) on the Lighthouse Letter Acuity charts and MNREAD charts were tested at 160 cm to ensure adequate resolution. To change the viewing distance, the test was paused and subjects were moved back from 100 to 160 cm.

### CSF Filters

**FIGURE 1:**
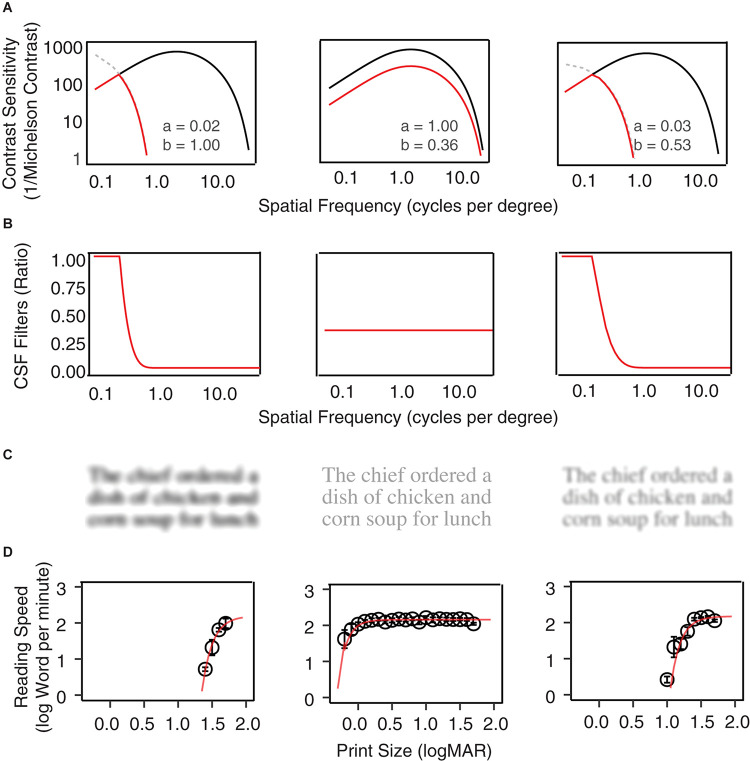
Examples of CSF filters. **(A)** Normal vision CSFs are represented with black curves, and the low vision CSFs are represented by red curves with horizontal and vertical scaling of the normal CSF. From left to right, the plots show examples of horizontal scaling, vertical scaling, and horizontal-plus-vertical scaling conditions. The scaling factors are listed in each plot. **(B)** The CSF filters are defined by the ratio between the low vision and normal vision CSF in **(A)**. **(C)** A MNREAD sentence filtered by the three CSF filters. **(D)** Reading speed (log word per minute) as a function of print size under the three conditions.

In Figure 1A, the black curve illustrates a normal CSF template, with the y-axis representing contrast sensitivity and x-axis representing spatial frequency. The CSF was constructed based on Barten’s simplified CSF formula (Equation 1, Barten, 1999, 2003). In Equation 1, *S_NV_(f)* is the contrast sensitivity at spatial frequency f, equivalent to the inverse of the corresponding Michelson contrast at threshold. There are two free parameters: the luminance (*L*) of the image and the angular area (*X*_0_^2^) of the picture area. The luminance was fixed as the mean luminance of the screen (150 cd/m^2^), and the image area was fixed as the angular area of the screen (33 × 19 deg^2^).

(1) $$S_{N\,V}\,\left(f\right)=\frac{5200\,e^{-0.0016\,f^{2}\,{\left(1+100/L\right)}^{0.08}}}{\sqrt{\left(1+\frac{144}{X_{0}^{2}}+0.64\,f^{2}\right)\times \left(\frac{63}{L^{0.83}}+\frac{1}{1-e^{-0.02\,f^{2}}}\right)}}$$

Low-vision CSF curves were created by shifting the normal CSF curve horizontally along the spatial frequency axis by factor *a* and vertically along the contrast sensitivity axis by factor *b* (Equation 2; Chung and Legge, 2016). The scaling corresponds to horizontal and vertical translations of the normal template in the log-log coordinates of Figure 1A. The red curves in Figure 1A provide three low-vision CSF examples of different combinations of horizontal and vertical scaling. Note that in some conditions the shifted low-vision sensitivities at lower spatial frequencies would exceed that of the normal vision (Figure 1A, gray dashed curves). To avoid this problem the low-vision CSF was clamped at the value of the normal CSF.

(2) $$S_{L\,V}\,\left(f\right)=\text{min}\,\left(b\,S_{N\,V}\,\left(\frac{f}{a}\right),S_{N\,V}\,\left(f\right)\right)$$

The CSF filter is constructed by computing the attenuation in spatial frequency components of the input image due to contrast sensitivity loss across the low-vision CSF relative to the normal CSF. It is defined as the ratio between a low-vision CSF and the normal CSF (Equation 3, Figure 1B).

(3) $$F\,\left(f\right)=\frac{S_{L\,V}\,\left(f\right)}{S_{N\,V}\,\left(f\right)}$$

The CSF filters can be applied to digital texts and pictures to simulate pattern visibility to the corresponding low-vision eyes. Specifically, the amplitude of the Fourier transform of the input image at each spatial frequency is multiplied by the corresponding value of the filter function to achieve spatial-frequency specific attenuation, and then an inverse Fourier transform is applied to create the filtered image. Figure 1C shows examples of a MNREAD sentence after filtering by three CSF filter conditions. Figure 1D shows the average reading curves under each of the three simulated conditions.

As an aside, we comment on a methodological difference in the implementation of the CSF filtering between the current study and the previous studies of Peli (1990) and Thompson et al. (2017). Specifically, in the two previous studies, a visual image was decomposed into a discrete set of frequency bands. A contrast threshold was then derived from the low-vision CSF for each frequency band and applied to the corresponding sub-image to completely eliminate the image contents with sub-threshold contrasts. This non-linear filtering approach is particularly suitable for the simulation of the appearance of complex images where local contrast plays a vital role in pattern perception. However, One issue with this approach is the noticeable artifacts (i.e., banding or ringing effects) generated in the filtered images due to the use of non-linear hard-thresholding. Although a solution has been proposed by Thompson et al. (2017) to minimize the artifacts, they are still visible and can be distracting in deciphering letters in text.

In the current study, we adopted an alternative linear approach, using a single-channel filter based on the CSF, that does not involve decomposing the entire frequency range into a discrete set of frequency bands and no explicit thresholding is performed. Our previous work has preliminarily validated this approach for simulating low-vision visibility (Lei et al., 2016). In this approach, the ratios of low-vision and normal-vision contrast sensitivities at all spatial frequencies spanning the CSFs were calculated as the filter to represent the loss of contrast sensitivities in low vision relative to normal vision. The filter was then used to linearly scale the spatial frequency contents of an image, such that each frequency component was attenuated by an amount that is commensurate with the relative loss in contrast sensitivity of a low-vision observer at that frequency. The linear approach results in filtered images of text that are virtually free of artifacts. Linear filtering is also simpler to implement with fewer parameter settings than needed for the sub-band thresholding implemented in the non-linear method.

### Simulated Low-Vision Conditions

A close association can be established between the scaling factors (a and b) used in the simulation of low vision and the corresponding visual acuity (VA) and contrast sensitivity (CS) values we intend to simulate. Briefly, VA provides an estimation of the high frequency cut-off of the corresponding CSF, and CS provides an estimation of the peak contrast sensitivity of the corresponding CSF. For people with low vision, the reductions in their VA and CS compared to the normal baselines can therefore be associated with the horizontal and vertical scaling factors. For purposes of our modeling, the normal baseline acuity was −0.24 logMAR, corresponding to the high-frequency cutoff of the normal CSF, and the normal baseline value for CS was 2.13 log units, corresponding to the mean Pelli-Robson score of our YN subjects (see footnotes in Table 1). Supplementary Appendix 1 describes the transformations relating the scaling factors *a* and *b* to measured values of VA and CS. The parameterization procedure is similar in logic to that of Thompson et al. (2017) but different in implementation due to the adoption of a different functional form for the CSF.

**TABLE 1:** Simulated low-vision conditions: scaling factors (a and b), expected VA and CS Reductions, expected VA and CS values, and measured VA and CS values (mean [standard deviation]) in the normally sighted young group.

**Filter**	**Horizontal scaling *a***	**Vertical scaling *b***	**Expected VA reduction**	**Expected CS reduction**	**Expected VA**	**Expected CS**	**Measured VA**	**Measured CS**
0*	1.000	1.000	0.00	0.00	−0.24^†^	2.13^‡^	−0.09 [0.02]	2.13 [0.01]
1	0.288	0.288	0.60	−0.54	0.36	1.59	0.28 [0.01]	1.58 [0.02]
2	0.157	0.157	0.90	−0.80	0.66	1.33	0.62 [0.01]	1.33 [0.02]
3	0.086	0.086	1.20	−1.06	0.97	1.07	0.97 [0.01]	0.99 [0.02]
4	0.048	0.048	1.51	−1.31	1.27	0.82	1.31 [0.02]	0.64 [0.02]
5	0.027	0.027	1.81	−1.54	1.57	0.59	1.72 [0.01]	0.10 [0.02]
6	0.250	1.000	0.60	−0.07	0.36	2.05	0.25 [0.02]	2.11 [0.02]
7	0.134	0.534	0.90	−0.31	0.66	1.81	0.56 [0.01]	1.80 [0.03]
8	0.072	0.288	1.20	−0.57	0.97	1.55	0.93 [0.02]	1.44 [0.02]
9	0.039	0.157	1.51	−0.83	1.27	1.29	1.27 [0.03]	0.95 [0.07]
10	0.022	0.086	1.81	−1.09	1.57	1.03	1.67 [0.02]	0.35 [0.04]
11	0.267	0.534	0.60	−0.27	0.36	1.86	0.26 [0.01]	1.91 [0.04]
12	0.144	0.288	0.90	−0.54	0.66	1.59	0.57 [0.01]	1.60 [0.03]
13	0.078	0.157	1.20	−0.80	0.97	1.33	0.96 [0.02]	1.23 [0.03]
14	0.043	0.086	1.51	−1.06	1.27	1.07	1.30 [0.02]	0.72 [0.03]
15	0.024	0.048	1.81	−1.31	1.57	0.82	1.68 [0.01]	0.27 [0.03]
16	0.314	0.157	0.60	−0.80	0.36	1.33	0.30 [0.02]	1.26 [0.07]
17	0.172	0.086	0.90	−1.06	0.66	1.07	0.65 [0.03]	1.01 [0.06]
18	0.096	0.048	1.20	−1.31	0.97	0.82	1.01 [0.02]	0.69 [0.05]
19	0.055	0.027	1.51	−1.54	1.27	0.59	1.40 [0.04]	0.36 [0.03]
20	0.032	0.016	1.81	−1.76	1.57	0.42	1.77 [0.01]	0.00 [0.00]
21	0.345	0.086	0.60	−1.06	0.36	1.10	0.35 [0.02]	1.07 [0.04]
22	0.193	0.048	0.90	−1.31	0.66	0.85	0.69 [0.01]	0.79 [0.04]
23	0.110	0.027	1.20	−1.54	0.97	0.62	1.01 [0.03]	0.64 [0.05]
24	0.064	0.016	1.51	−1.76	1.27	0.40	1.38 [0.05]	0.30 [0.04]
25	0.038	0.010	1.81	−1.96	1.57	0.14	1.57 [0.01]	0.15 [0.00]
26	0.439	0.027	0.60	−1.54	0.36	0.58	0.50 [0.04]	0.62 [0.07]
27	0.256	0.016	0.90	−1.76	0.66	0.36	0.85 [0.04]	0.47 [0.05]
28	0.154	0.010	1.20	−1.96	0.97	0.16	1.31 [0.07]	0.29 [0.07]
29	0.033	0.534	1.51	−0.55	1.27	1.61	1.32 [0.03]	1.50 [0.04]
30	0.018	0.288	1.81	−0.81	1.57	1.35	1.63 [0.02]	0.77 [0.03]
31	0.125	1.000	0.90	−0.16	0.66	1.96	0.64 [0.02]	2.06 [0.04]
32	0.063	1.000	1.20	−0.29	0.97	1.85	0.97 [0.02]	1.93 [0.02]
33	0.031	1.000	1.51	−0.45	1.27	1.67	1.33 [0.01]	1.52 [0.08]
34	0.016	1.000	1.81	−0.63	1.57	1.49	1.55 [0.01]	0.69 [0.09]
35	1.000	0.355	0.05	−0.45	−0.19	1.65	−0.12 [0.01]	1.70 [0.03]
36	1.000	0.178	0.09	−0.75	−0.15	1.35	−0.07 [0.02]	1.43 [0.03]
37	1.000	0.089	0.14	−1.04	−0.10	1.06	−0.02 [0.03]	1.10 [0.03]
38	1.000	0.045	0.20	−1.34	−0.04	0.76	0.07 [0.03]	0.88 [0.03]
39	1.000	0.022	0.27	−1.63	0.03	0.47	0.17 [0.04]	0.58 [0.03]
40	1.000	0.011	0.36	−1.90	0.13	0.20	0.45 [0.09]	0.36 [0.04]

*Filter 0 represents no filtering condition.

*^†^The baseline acuity value was directly obtained from the cut-off spatial frequency (51.9 cpd) of the normal CSF function, using Equation 11 in Supplementary Appendix 2.*

*^‡^The baseline Pelli-Robson contrast sensitivity value obtained from the peak of the normal CSF function is 2.5 log units, which exceeds the test capacity of the Pelli-Robson chart and therefore was normalized to the baseline value of our subject pool.*

**FIGURE 2:**
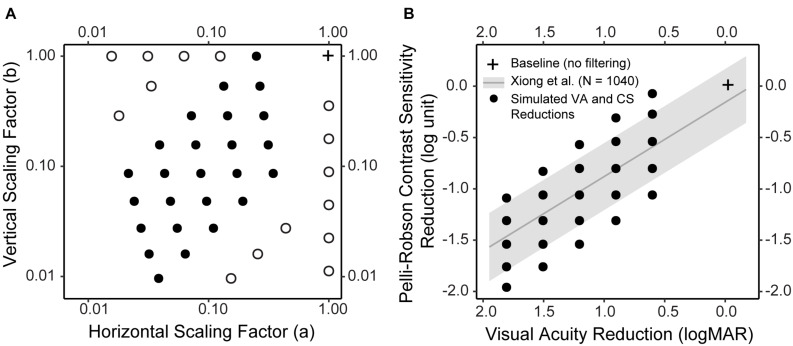
Simulated low vision conditions. **(A)** The horizontal and vertical scaling factors of the forty simulated low vision conditions. Twenty-five conditions (solid dot) were determined so that the reductions in CS and VA would closely resemble the empirical distribution (see **B** for details). Fifteen conditions (empty dot) were boundary conditions. **(B)** The empirical distribution of CS reduction as a function of VA reduction, both compared to Age-adjusted normal baselines, in a large sample of subjects (*N* = 1,040) with various vision conditions (adapted from Xiong et al., 2020). The gray line represents the regression line, and the gray ribbon represents the 95% confidence interval around the regression line. The black solid dots represent the CS and VA reductions in the 25 simulations. Note that in **B** the VA axis was reversed, i.e., from large to small, to visualize the correspondence between **A** and **B**. In both figures, the cross represents the baseline condition without filtering.

Forty hypothetical low-vision conditions were simulated using different combinations of horizontal and vertical scaling. The scaling factors *a* and *b* used in the forty hypothetical low-vision conditions are listed in Table 1 and illustrated in Figure 2A.

Twenty-five of the low-vision conditions (Figure 2A, filled circles; Table 1, Filter 1–25) were determined based on the empirical relationship between VA and CS (adapted from Xiong et al., 2020). Specifically, across a large sample of subjects (*N* = 1,040) including those with normal ocular health and various ocular pathologies, the reductions in VA and CS compared to normal baselines were significantly correlated following a linear relationship (the regression line and confidence intervals are presented in Figure 2B). We first determined five hypothetical low-vision conditions corresponding to 0.6, 0.9, 1.2, 1.5, and 1.8 logMAR reductions compared to normal VA. For each level of VA reduction, five levels of CS reductions were determined by steps of 0.25 log unit, with the middle level centered approximately at the regression line (Figure 2B, black dots). The remaining fifteen low-vision conditions (Figure 2A, open circles; Table 1, Filter 26–40) were retrospectively included to supplement the boundary conditions.

### Procedure

All tests were conducted under binocular viewing. Each YN subject was tested with a baseline condition where no filtering was applied, and between 10 and 16 simulated low-vision conditions. VA, CS, and reading performance were measured under each condition, using digital versions of the Lighthouse Letter Acuity charts, Pelli-Robson Contrast Sensitivity charts and the MNREAD charts, respectively. The ON, Mac, and Non-Mac groups also completed the three tests, under the no filtering condition only.

All the testing and scoring followed the standard protocols for the tests. VA was scored on a letter-by-letter basis with each letter worth 0.02 logMAR (Ferris et al., 1982), and CS scored as the log value of the lowest contrast at which subjects can correctly report at least 2 letters in a triplet (Pelli et al., 1988).

Reading speed in log word per minute (log wpm) was obtained at each tested print size. Reading speed as a function of print size was fitted with a function (Equation 4) by non-linear mixed-effects (NLME) modeling, in which subject variations were modeled as random effects (Cheung et al., 2008).

(4) $$R\,e\,a\,d\,i\,n\,g\,S\,p\,e\,e\,d=m\,r\,s\times \left(1-e^{\left(-e^{l\,r\,c}\times \left(P\,r\,i\,n\,t\,S\,i\,z\,e-x\,i\,n\,t\right)\right)}\right)$$

where mrs is the plateau of the reading curve, lrc is the slope of the reading curve, and xint is the intercept of the reading curve with x-axis. Three standard reading indices were derived from each fitted curve:

• Maximum reading speed: the fastest reading speed subjects can achieve. Calculated as the asymptote of the fitted exponential curve.
• Critical print size: the smallest print size yielding the maximum reading speed. Calculated as the print size corresponding to a reading speed of 90% of the maximum reading speed.
• Reading acuity: the smallest print size that can just be read. Reading acuity = smallest print size attempted + number of errors × 0.01.

Table 2 provides a summary of the VA, CS, and reading indices for each group.

**TABLE 2:** Age, vision status, and reading performance of the young normal (YN), older normal (ON), non-macular (Non-Mac) and macular (Mac) groups (mean [standard deviation]).

**Groups**	**Age (years)**	**VA (logMAR)**	**CS (log unit)**	**Maximum reading speed (wpm)**	**Critical print size (logMAR)**	**Reading acuity (logMAR)**
YN*	20.5 [3.6]	−0.09 [0.11]	2.13 [0.07]	2.20 [0.06]	0.21 [0.10]	−0.14 [0.07]
ON	68.0 [5.0]	0.00 [0.11]	2.02 [0.11]	2.17 [0.05]	0.26 [0.12]	−0.05 [0.10]
Non-Mac	56.7 [16.1]	0.82 [0.40]	0.96 [0.52]	2.06 [0.16]	1.35 [0.82]	0.71 [0.41]
Mac	72.9 [16.7]	0.64 [0.31]	1.16 [0.33]	2.07 [0.21]	1.49 [0.90]	0.71 [0.40]

*For the YN group, the listed VA, CS and reading indices were under baseline condition with no filtering.

### Statistical Analysis

The statistical analyses were performed using the R package (R Core Team, 2018). When examining the validity of simulating reduced VA and CS, two Linear Mixed Effects (LME) models (Pinheiro and Bates, 2000) were conducted on the VA or CS values, with value types (expected and measured) and filter conditions as fixed factors and subject as a random effect. In addition, we used the test-retest reliabilities (95% coefficient of repeatability) of VA (0.20 logMAR) and CS (0.30 log unit) for low vision as the criterion of clinically significant difference (Kiser et al., 2005).

Three LME models were fit to describe the impact of simulated VA and CS reduction on reading performance (maximum reading speed, critical print size, reading acuity), respectively. Specifically, the models treated the reading indices as the dependent variable, expected VA and CS as the fixed effect factors, and subject and filter as random effects. For maximum reading speed, an additional Non-linear Mixed Effect (NLME) model (Bates et al., 2015) was fit to further quantify the impact of simulated CS reduction.

Lastly, when examining the validity of the simulation in predicting reading performance in low-vision subjects and older control subjects, again LME models were fit to compare the predicted and actual reading performance (maximum reading speed, critical print size and reading acuity). The models treated the reading indices as the dependent variable, condition (predicted vs. actual values) and group (Non-Mac, Mac, and ON) as fixed effects, and subject as a random effect.

For all the LME analyses described above, the significance of each fixed factor was examined by the ANOVA function in the “lme4” package. *Post hoc* analysis was performed with Bonferroni corrections (“emmeans” package, Piepho, 2004). *p*-values smaller than 0.05 were considered statistically significant.

## Results

### Validity: Simulating Reduced Acuities and Contrast Sensitivities in Normally Sighted Young Subjects

First, we asked if the CSF filter with certain parameters would actually yield the expected VA and CS scores for normally sighted young subjects. For example, for parameter values *a* = 0.29 and *b* = 0.29 (Table 1, Filter 1), would the filtered versions of the acuity and contrast sensitivity charts yield the expected test scores of 0.36 logMAR and 1.59 log unit? This validation is important to confirm that two key assumptions underlying our simulation are valid—first, that the use of a horizontally and vertically shifted normal CSF template is a good approximation of a low-vision reader’s CSF, and second, that clinical measures of visual acuity and contrast sensitivity can be used to calculate the horizontal and vertical shifts.

**FIGURE 3:**
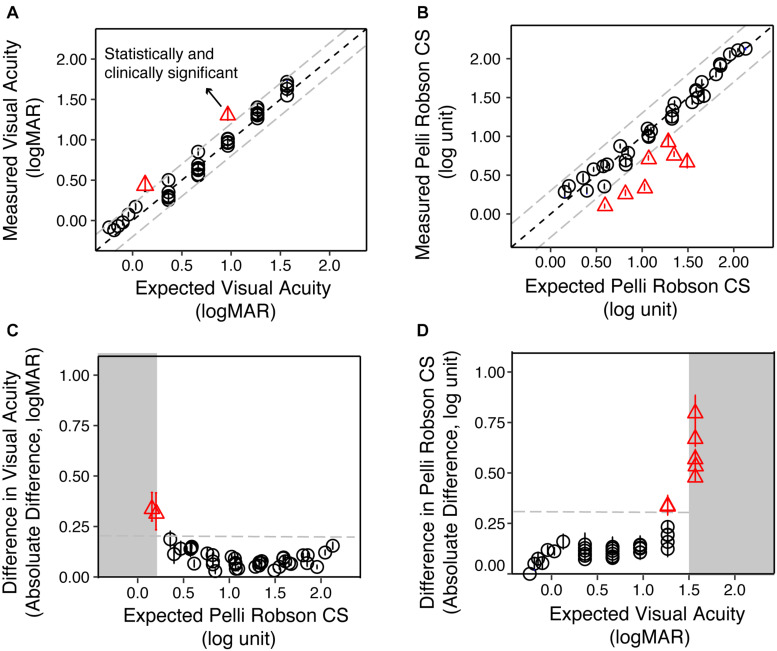
The validity of simulating reduced contrast sensitivity and visual acuity in the Young Normal (YN) group. **(A)** The measured VA as a function of the expected VA. The circles represent the group average of each condition, and the error bars represent the standard errors. The red triangles represent conditions where the differences between the measured and expected values were statistically (*p* < 0.05) and clinically significant (>0.20 logMAR). **(B)** The measured CS as a function of the expected CS. The red triangles represented conditions where the differences between the measured and expected values were statistically (*p* < 0.05) and clinically significant (>0.30 log unit). **(C)** The difference between measured and expected VA as a function of the expected CS. When the expected CS was close to or worse than 0.15 log unit, the difference between measured and expected VA was the largest. **(D)** The difference between measured and expected CS value as a function of the expected VA. When the expected VA values were close to or exceeded 1.50 logMAR, the difference between measured and expected CS were the largest. In all four plots, the gray dashed lines represent the clinically significant difference in low vision (0.20 logMAR for VA and 0.30 log unit for CS), respectively.

Figures 3A,B are scatter plots of the measured VA and CS vs. the expected values from the simulation for the YN group. The dots represent the group average for each low vision simulation, and the solid line represents the equality line.

We used the test-retest reliability of VA and CS (0.20 logMAR and 0.30 log unit, respectively) as criteria for clinically significant differences (Kiser et al., 2005). The difference between the measured and expected VA ranged from −0.11 to 0.35 logMAR, with a median of 0.05 logMAR. LME analysis on VA showed significant main effect of value type [expected and measured; *F*(1, 852) = 141.31, *p* < 0.001], filter conditions [*F*(40, 774) = 2098.15, *p* < 0.001], and an interaction between them [*F*(40, 852) = 19.04, *p* < 0.001]. *Post hoc* analysis with Bonferroni corrections showed that only two conditions showed a significant difference (*p* < 0.05) larger than 0.20 logMAR (Figure 3A, red triangles).

The difference between the measured and expected CS ranged from −0.81 to 0.16 log unit, with a median of −0.03 log unit. LME analysis on CS showed significant main effect of value type [expected and measured; *F*(1, 829) = 278.05, *p* < 0.001], filter conditions [*F*(40, 485) = 885.76, *p* < 0.001], and an interaction between them [*F*(40, 829) = 38.73, *p* < 0.001]. *Post hoc* analysis with Bonferroni corrections showed that seven conditions had significant differences (*p* < 0.05) that were larger than 0.30 log unit (Figure 3B, red triangles).

We asked whether these deviant points are associated with an interaction between poor acuity or contrast sensitivity. We plotted the difference between the expected and measured VA as a function of the expected CS (Figure 3C), and found that the two conditions that reached clinical significance both had the lowest expected CS (0.15 and 0.20 log unit). Similarly, the seven conditions that reached clinical significance in CS all had expected VA close to or larger than 1.5 logMAR (Figure 3D). LME modeling confirmed this mutual impact between VA and CS. The difference between measured and expected VA increase as CS worsens [*F*(1, 43) = 14.07, *p* < 0.001] and vice versa [*F*(1, 39) = 34.40, *p* < 0.001].

### Validity: Simulation of Reading Performance

Do the simulated acuity and contrast sensitivity reductions show similar impacts on reading as real low vision? If this is the case, the real and simulated low-vision subjects with equivalent VA and CS should have similar reading performance.

**FIGURE 4:**
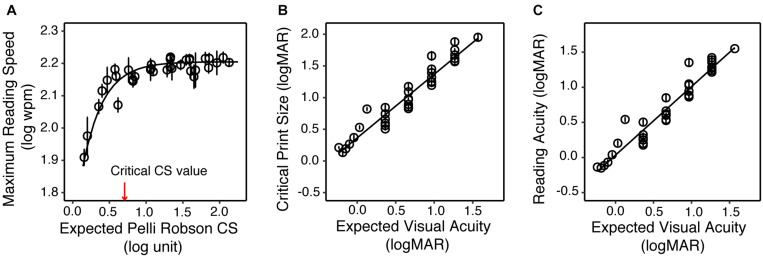
The impact of simulated acuity and contrast sensitivity reduction on the reading performance of the YN group. **(A)** Maximum reading speed as a function of the expected CS. The red arrow represents the critical CS for a maximum reading speed of at least 90% of the maximum reading speed for normal vision (asymptote). **(B)** Critical print size as a function of the expected VA. **(C)** Reading acuity as a function of the expected VA. In all three plots the circles represent the group average of each simulated low vision condition, and the error bars represent standard errors.

Figure 1D illustrated how reading speed changed with print size when text images were filtered with three sample filters. To quantify the impact of the simulated VA and CS on reading, we built LME models across all simulation conditions for the YN subjects on the three reading indices, with the expected VA and CS as predictors. Maximum reading speed was only significantly affected by CS [*F*(1, 32) = 30.83, *p* < 0.001], and it was mostly unaffected until the simulated CS was very low (Figure 4A). We quantified the impact of CS on the maximum reading speed by an exponential function, which showed that when CS dropped to 0.69 log unit, the maximum reading speed only decreased by 10%. VA and CS were both significant predictors for critical print size and reading acuity (all *p* < 0.001). VA alone explained 88% and 90% of the variations in the critical print size and reading acuity, respectively (Figures 4B,C). With the addition of CS, they explained 95% of the variance in the critical print size and 96% in the reading acuity. The parameters of the three regression models are provided in Table 3.

**TABLE 3:** Regression models on maximum reading speed, critical print size, and reading acuity with VA and CS as predictors.

	**VA**	**CS**	***R*^2^**	**Regression model**
Maximum reading speed	*F*(1, 35) = 0.42, *p* = 0.52	*F*(1, 32) = 30.83, *p* < 0.001	0.25	Maximum reading speed=2.20 × (1– exp(−3.56 × (CS+0.40)))
Critical print size	*F*(1, 38) = 1333.47, *p* < 0.001	*F*(1, 34) = 88.78, *p* < 0.001	0.95	Critical print size=0.65 + 0.95 × VA - 0.21 × CS
Reading acuity	*F*(1, 35) = 1111.36, *p* < 0.001	*F*(1, 37) = 73.14, *p* < 0.001	0.96	Reading acuity=0.30 + 0.95 × VA - 0.21 × CS

**FIGURE 5:**
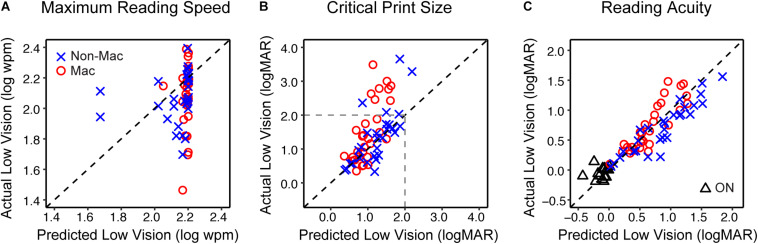
The validity of simulating the reading performance of low vision individuals. **(A)** The comparison of the maximum reading speed between actual low vision and predicted low vision from the simulation. The blue crosses and red circles represent the Non-Mac and Mac groups, respectively. **(B)** Comparison of the critical print size for actual and predicted low vision from the simulation. Note that critical print sizes larger than 2.0 logMAR (dashed lines) may be unreliable due to limitations on the tested print size range. **(C)** Comparison of reading acuity for actual and simulated low vision from the simulation. Comparisons between actual and simulated reading acuity of the Older Normal (ON) group are also shown (black triangles). Note that for the ON group this comparison was only conducted on reading acuity.

We then tested if the models we derived from simulation can reasonably predict the reading performance of subjects with actual low vision. Specifically, the reading indices of actual low-vision subjects were obtained from the curves fitted to their reading data (individual data are provided in Supplementary Appendix 2), and the predicted reading indices were obtained by entering their VA and CS into the regression models in Table 3. Figure 5 shows scatterplots of the actual versus predicted reading indices for each low-vision subject.

The predicted maximum reading speed (Figure 5A) was faster than the actual value in both Non-Mac and Mac groups, by an average of 0.07 log wpm (equivalent to 17%, *p* = 0.058) and 0.12 log wpm (equivalent to 32%, *p* < 0.001), respectively. The predicted critical print size (Figure 5B) was in close agreement with the actual values in the Non-Mac group (*p* = 0.27), but it was significantly smaller than the actual values in the Mac group (*p* < 0.001). Large deviations in the critical print size mostly appeared when the actual critical print size values were larger than 2.0 logMAR. These deviations may be due to the fact that under severe low-vision simulation conditions, the tested print size (−0.3 to 1.7 logMAR) was not sufficient to reflect the plateau of the reading curve, therefore the fitted curve may have yielded an unreliable estimation of critical print size. Within the 2.0 logMAR limit, the predicted critical print sizes were not significantly different from the actual values in both groups (*p* = 0.13 for the Non-Mac group, and *p* = 0.15 for the Mac group). The predicted reading acuity (Figure 5C) was in close agreement with the actual values in the Mac group (*p* = 0.13), and was slightly larger than the actual value in the Non-Mac group by an average of 0.16 logMAR (*p* < 0.001).

### Consideration of Age as an Additional Parameter for Low-Vision Simulation

Many low-vision conditions are age related, as shown by the age and pathology distributions of our low-vision sample. Therefore, we considered whether age should be included as an additional parameter to fine tune the simulation. To answer this question, we included a group of older subjects with normal ocular health (ON group).

Compared to the YN group, the ON group had a significantly larger value of VA by 0.09 logMAR (*p* = 0.017), and were lower in CS by 0.11 log units (*p* = 0.002). When comparing their reading performance with the YN group under the unfiltered condition, the ON group showed lower maximum reading speed, and higher logMAR values of critical print size and reading acuity, but only reading acuity reached significance (by 0.09 logMAR, *p* = 0.005). However, the reduced VA and CS in the ON group were sufficient to explain the age-related change of the reading acuity. When individual subject’s VA and CS values were entered into the regression model in Table 3, the predicted reading acuity was not significantly different from the actual reading acuity of the ON group (*p* = 0.12, Figure 5C). This means that we found no additional age effect on reading, once acuity and contrast sensitivity are taken into account.

## Discussion

Digital images of test letters and text were constructed based on the CSF filtering principle, to simulate low vision with various combinations of acuity and contrast sensitivity reduction. We examined the validity of this simulation by attempting to replicate low-vision performance by testing normally sighted subjects with test-chart letters and text reading. Regarding visibility, we found that our simulation reproduced the desired visual acuity and contrast sensitivity we intended to simulate in normally sighted young subjects. Regarding reading, we found that the simulation overestimated the maximum reading speed but provided a good estimate of critical print size and reading acuity, for real low-vision individuals with corresponding acuity and Pelli-Robson contrast sensitivity.

There has been increasing interest in estimating low-vision CSFs from clinical measures of acuity and contrast sensitivity (Chung and Legge, 2016; Thurman et al., 2016; Thompson et al., 2017). Our simulation of low-vision visibility rests on a simple model in which images are filtered by shifted versions of the normal CSF. The shifts along the log spatial frequency and log contrast sensitivity axes are related to clinical measures of letter acuity and contrast sensitivity by equations described in Supplementary Appendix 1.

The first step in validating the simulation was to verify that the filtered images of letter charts would produce the expected values of reduced acuity and contrast sensitivity when viewed by normally sighted subjects. We simulated forty low-vision conditions based on the empirical distributions of VA and CS across a large sample of subjects with normal vision and vision pathologies (Xiong et al., 2020). We found that when simulating low-vision conditions with acuities better than 1.5 logMAR (approximately 20/630) and contrast sensitivities above 0.15 log unit, the measured acuities and contrast sensitivities closely matched the expected values. For low-vision conditions outside these boundaries, the simulation had poorer performance. It is noteworthy that Pelli-Robson letter size (2 × 2 inches) subtends 2.91 degrees (1.54 logMAR) at a typical viewing distance of 1m used for low vision. This print size is difficult to recognize for individuals whose acuity is 1.5 logMAR or worse, even if they have good contrast sensitivity. This may explain the upper bound of logMAR acuity for our simulation, and indicate that in clinical practice the viewing distance of Pelli-Robson letters should be reduced for patients whose acuity is worse than 1.5 logMAR (Njeru et al., 2021).

We then asked if this simulation generalizes to more interesting real-world stimuli such as digital text or graphics. We compared the reading performance of real low-vision subjects with normally sighted subjects who read under simulated low-vision conditions with equivalent VA and CS. The simulation overestimated the maximum reading speed. The lack of correlation between maximum reading speed and acuity and the weak correlation between maximum reading speed and contrast sensitivity are consistent with previous findings in normal and low-vision reading (Legge et al., 1987; Rubin and Legge, 1989). It is likely that visual factors other than acuity and contrast sensitivity, e.g., visual field loss or unstable reading eye movements (Fletcher et al., 1999; Crossland et al., 2004; Calabrèse et al., 2014), may have detrimental effects on maximum reading speed.

The current simulation only considered acuity and contrast sensitivity reductions associated with low vision, but other visual or non-visual factors might be incorporated to improve the simulation. We examined whether an age adjustment should be included to account for the slight decline of reading performance in older age (Owsley, 2016; Calabrèse et al., 2016). We also compared the validity of the current approach in simulating low vision with or without central vision disturbance.

We found that the simulation based on acuity and contrast sensitivity reductions was sufficient to account for the decrements in reading in our older subjects. However, differences were shown between the Mac and Non-Mac groups, with the overestimations of the simulation for the reading indices being more prominent in the Mac group. These differences are consistent with the adverse impact of central field loss on reading that has been reported in earlier studies. Legge et al. (1985) found that low-vision subjects with central field loss showed slower peak reading speeds than acuity-matched subjects with remaining central vision. Crossland et al. (2004) found that subjects with macular diseases showed impaired fixation stability when reading texts. Therefore, although the visibility-based simulation can adequately replicate the reading performance of the majority of our low-vision subjects, including central field status as a third factor might improve the validity of the simulation.

**FIGURE 6:**
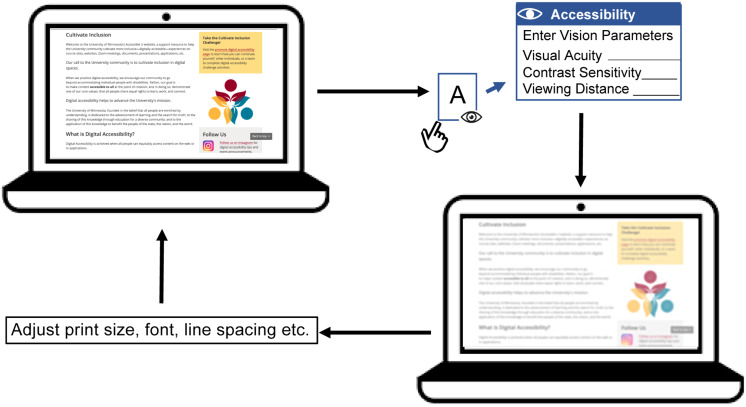
A flowchart illustrating the conceptual design of an accessibility checker for web pages. A web page with text, pictures and links is presented on a laptop screen. The accessibility checker functions as a plug-in for the web browser, which can be initiated by the user. Once initiated, the accessibility checker requests user input including acuity, contrast sensitivity (if available) and preferred viewing distance. The accessibility checker then transforms the web page to demonstrate visibility for the corresponding low-vision condition. This simulation will show an eye-care clinician, family member or web designer how visible the web content is expected to be for the person with low vision. Lastly, the user can adjust the display properties such as overall zoom or print size, font, line spacing etc. to make the page more accessible. The user can repeat this process until the web page appears accessible under the corresponding low vision condition.

Digital simulation makes it possible to visualize the information available to a person with low vision. Our study examined the feasibility of utilizing clinically measured acuity and contrast sensitivity to estimate low-vision CSFs across a wide range of low-vision conditions for the purpose of simulating the visibility of image features. What practical value might such a simulation have? Such digital simulation could serve as an “accessibility checker” in the development of architecture and reading related products, and assist people with low vision in choosing optimal reading configurations. Lei et al. (2018) and Thompson et al. (2021) have used similar CSF-based filtering methods to predict the visibility of architectural hazards for people with specified levels of reduced acuity and contrast sensitivity. In the context of reading displays for low vision, it may be possible to construct a web-based accessibility checker for low vision to predict whether a particular combination of print size, font and viewing distance would be legible for someone with specified acuity and contrast sensitivity. Such simulation could also be valuable for educational purposes, to help people with normal vision better understand constraints on visual performance due to low vision. Figure 6 is a flowchart illustrating how an “accessibility checker” might be used for web pages, potentially as a plug-in for a web browser. When initiated by the user, who might be an eye-care clinician, web designer or family member of a low vision reader, the accessibility checker would take as input a potential user’s acuity, contrast sensitivity and desired viewing distance. If contrast sensitivity is not known, an estimate can be made based on the linear relationship between acuity and contrast sensitivity (Xiong et al., 2020). The accessibility checker will then transform the appearance of the web page to simulate visibility of the screen features for the corresponding low-vision condition. Lastly, the user can adjust the overall zoom or properties (e.g., print size, font, line spacing etc.) of the web page to make it more accessible to the potential low-vision reader. Clinicians can also use this accessibility checker to examine whether the icons and texts of operating systems are accessible for a particular low vision patient. These examples refer to the appearance a single page or website might appear in a static view. In our reading test, the processing time of sentence transformation ranged from 0.5 to 0.7 s. This processing time is likely to be acceptable for evaluating the appearance of static text or other static web content. To simulate the appearance of dynamic content, such as web videos, faster processing would be necessary.

## Data Availability Statement

The raw data supporting the conclusions of this article will be made available by the authors, without undue reservation.

## Ethics Statement

The studies involving human participants were reviewed and approved by the University of Minnesota Institutional Review Board. The patients/participants provided their written informed consent to participate in this study.

## Author Contributions

Y-ZX and GEL designed the research. Y-ZX and QL performed the research. Y-ZX analyzed the data. Y-ZX, GEL, and QL wrote and revised the manuscript. AC provided editorial suggestions. All authors approved the final version.

## Conflict of Interest

The authors declare that the research was conducted in the absence of any commercial or financial relationships that could be construed as a potential conflict of interest. The reviewer AC declared a past co-authorship with several of the authors GEL, AC, and Y-ZX to the handling Editor.
