# Supplementary material for: Simulating Visibility and Reading Performance in Low Vision
Source: Front Neurosci. 2021 Jul 5;15:671121. doi: 10.3389/fnins.2021.671121 (PMC8287255; doi:10.3389/fnins.2021.671121)
Supplement: Supplementary file 1 [file Data_Sheet_1.docx]

Supplementary Material

# Appendix 1. Conversion between the Horizontal and Vertical Scaling Factors of Low Vision CSF and the Clinically Measured Visual Acuity and Pelli-Robson Contrast Sensitivity

To build a CSF filter to simulate the visibility of texts and pictures for a particular person with low vision, the corresponding horizontal scaling factor *a* and vertical scaling factor *b* can be estimated based on the low vision person’s VA and CS values. For any known pairs of scaling factors *a* and *b*, the corresponding VA and CS values can also be estimated accordingly.

## Estimating scaling factors based on clinically measured VA and CS values

Step 1: Estimating vertical scaling factor

CS measured by the Pelli-Robson contrast sensitivity chart is a convenient measure of the peak contrast sensitivity, expressed as the opposite of the log of Weber contrast at threshold. For a normally sighted individual with CS_NV_, and a low vision individual with CS_LV_, the ratio between their Weber contrast ($r_{CW}$) is:

$r_{CW}= \frac{{10}^{-{CS}_{LV}}}{{10}^{{-CS}_{NV}}}= {10}^{{CS}_{NV}- {CS}_{LV}}$; Equation 1

However, contrast sensitivity on the contrast sensitivity function (CSF) are expressed as the reciprocal of the Michelson contrast. For a normal CSF with a peak contrast sensitivity of *PCN*, the Michelson contrast ($C_{M\_NV}$) and the corresponding Weber contrast ($C_{W\_NV}$) are:

$C_{M\_NV}= \frac{1}{PCN};$ $C_{W\_NV}= \frac{2C_{M\_NV}}{1+ C_{M\_NV}}= \frac{2}{PCN+1}$; Equation 2

When a vertical scaling factor *b* is applied to the normal CSF, the change in Weber contrast ($r_{CW}$) can also be obtained by:

$r_{CW}= \frac{\frac{2}{b\times PCN+1}}{\frac{2}{PCN+1}}= \frac{PCN+1}{b\times PCN+1};$ Equation 3

Joining Equation 1 and Equation 3, we have:

$b=\left( 1+\frac{1}{PCN} \right){10}^{{CS}_{LV}-{CS}_{NV}}- \frac{1}{PCN}$; Equation 4

Notice that when PCN is large, as in our normal CSF template (PCN = 732), Equation 4 can be simplified as:

$b={10}^{{CS}_{LV}-{CS}_{NV}}$; Equation 5

Step 2: Estimating horizontal scaling factor

VA is a convenient measure of the high frequency resolution limit on the CSF curve, or the cut-off spatial frequency. As shown in Figure 1a, both horizontal and vertical scaling induce changes in the cut-off spatial frequency.

The low vision cut-off frequency ${SF}_{LV}$ can be estimated from VA based on the standard assumption that SLOAN letters with angular size 0 logMAR corresponds to grating spatial frequency of 30 cpd (﻿Regan et al., 1981).

${SF}_{LV}=30\times{10}^{-VA};$ Equation 6

The vertical scaling factor *b* has been obtained in Step 1. The contribution of the vertical scaling to the cut-off frequency (${SF}_{b}$) can be obtained by solving:

$b\times S_{NV}\left( {SF}_{b} \right)=1;$ Equation 7

The contribution of horizontal scaling can thus be obtained by contrasting ${SF}_{b}$ and ${SF}_{LV}$:

$a= \frac{{SF}_{LV}}{{SF}_{b}}= \frac{30*{10}^{-VA}}{{SF}_{b}}$; Equation 8

## Estimating VA and CS values by scaling factors

Step 1: Estimating CS value

Based on Equation 4, when *b* is known, the CS_LV_ can be obtained by:

${CS}_{LV}={CS}_{NV}+log10(\frac{b\times PCN+1}{PCN+1})$; Equation 9

Note that with some combinations of horizontal and vertical scaling, the corresponding low vision CSF might exceed the normal CSF at low spatial frequencies (Figure 1a). In such cases, the low-vision CSF was clamped to the normal CSF. If the peak contrast sensitivity of the low-vision CSF is within the “clamped” range, the actual low-vision peak contrast sensitivity PCL would be lower than $b\times PCN$.

Step 2: Estimating VA value

The new cut-off spatial frequency${SF}_{LV}$ can be obtained by solving:

$bS_{NV}\left( \frac{{SF}_{LV}}{a} \right)= 1;$ Equation 10

The VA value can then be estimated by:

$VA=-log10(\frac{{SF}_{LV}}{30})$ ; Equation 11

Regan D, Raymond J, Ginsburg AP, Murray TJ. Contrast sensitivity, visual acuity and the discrimination of Snellen letters in multiple sclerosis. Brain. 1981;104: 333–350.

# Appendix 2. Reading Curves of the Low-Vision Subjects.

**
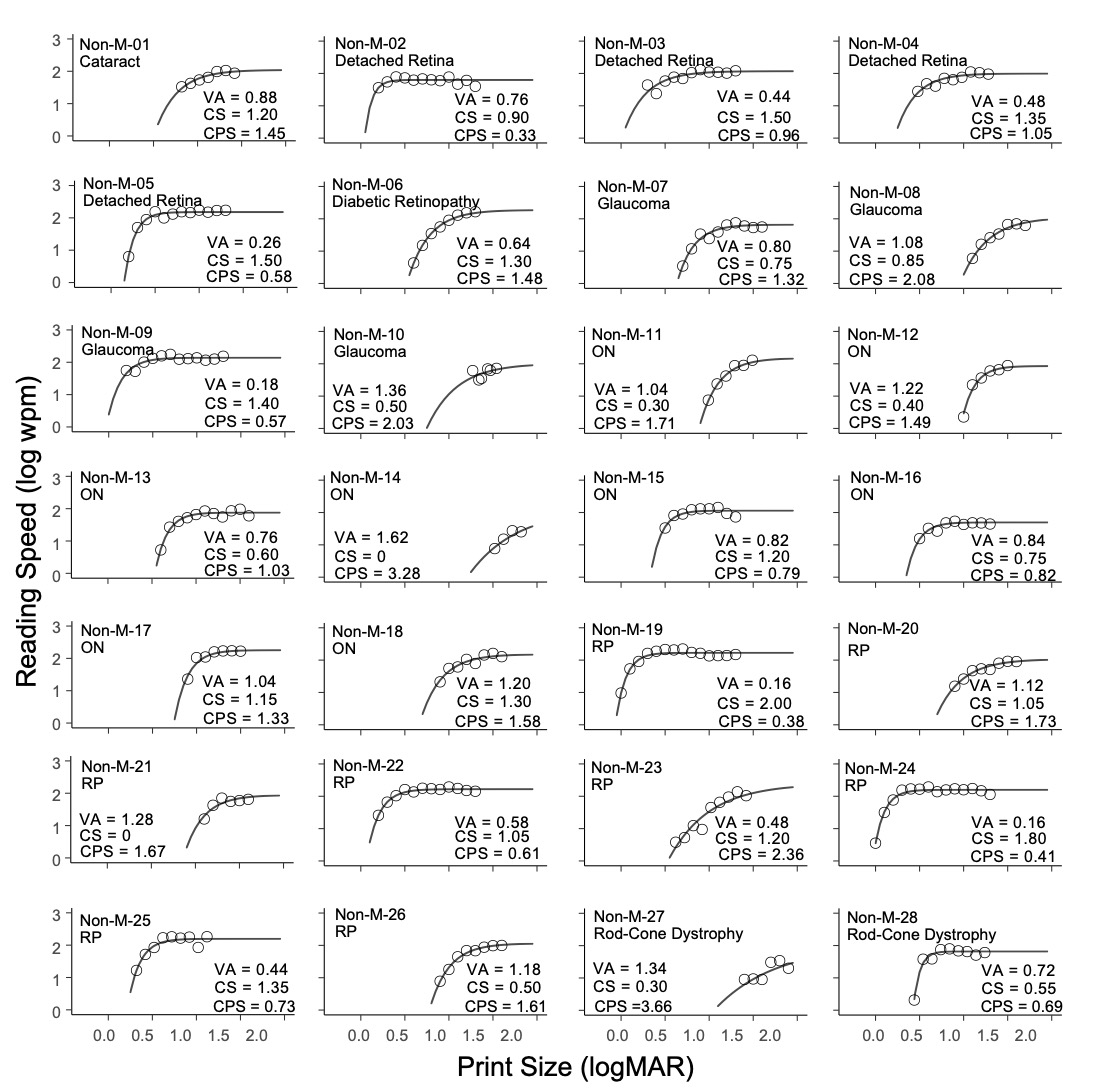
Supplementary Figure 1.** Individual reading curves and diagnoses for the Non-Mac group. Visual acuity, Pelli-Robson contrast sensitivity, and critical print size are also annotated in each plot. RP: Retinitis Pigmentosa. ON: Optic Neuropathy.


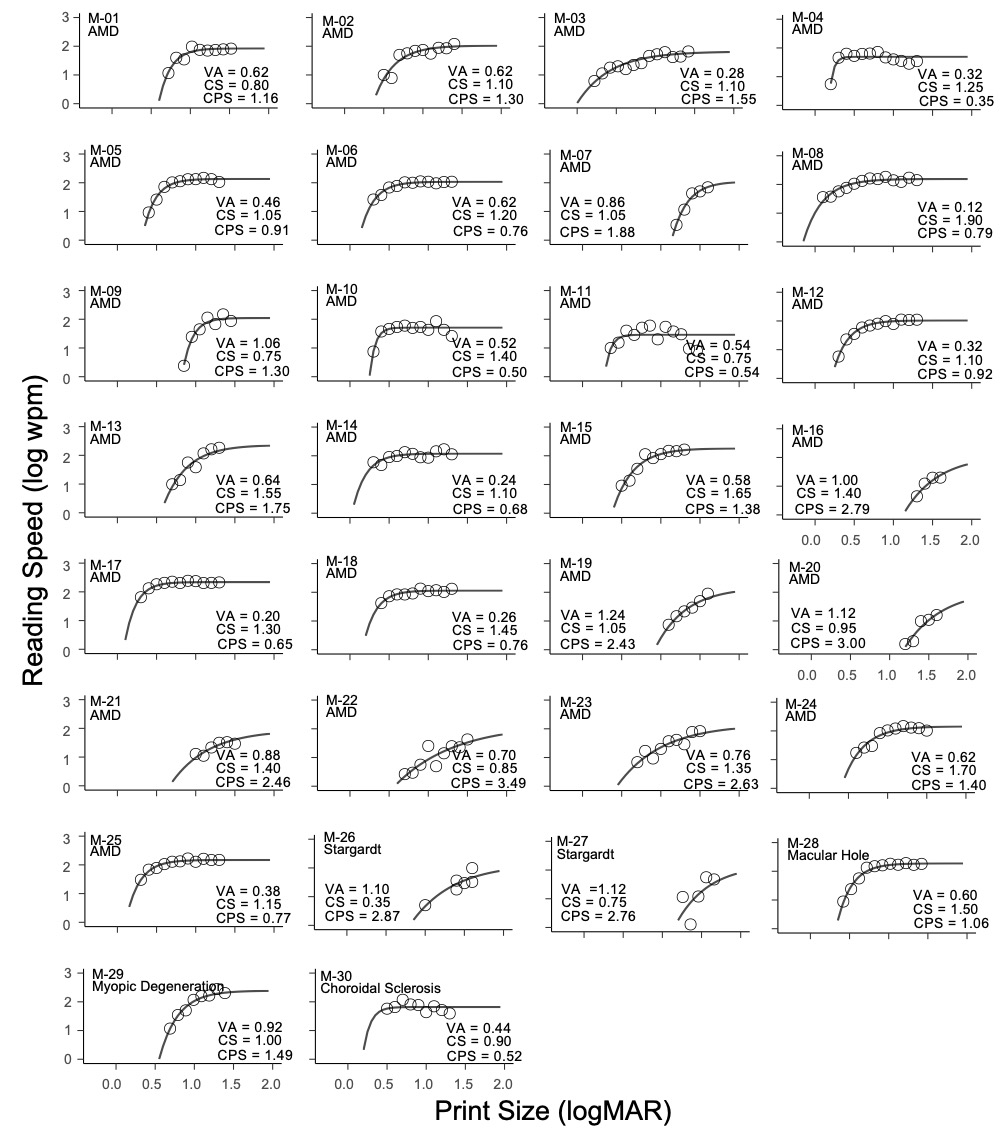


**Supplementary Figure 2.** Individual reading curves and diagnoses for the Mac group. Visual acuity, Pelli-Robson contrast sensitivity, and critical print size are also annotated in each plot. AMD: Age-related Macular Degeneration.
